# Radiographic assessment of frontal sinus morphology for gender determination using paranasal sinus view

**DOI:** 10.6026/973206300222588

**Published:** 2026-04-30

**Authors:** Abhay Deep Singh, Priyadershini A Rangari, Souryakant Varandani, Subash Chandra Nayak, Neelam Singh, Manju J

**Affiliations:** 1 Department of ENT, Shri Ram Murti Smarak Institute of Medical Sciences, Bareilly, Uttar Pradesh, India; 2 Department of Dentistry, Nandkumar Singh Chouhan Government Medical College, Khandwa, Madhya Pradesh, India; 3 Department of Pharmacology, Nandkumar Singh Chouhan Government Medical College, Khandwa, Madhya Pradesh, India; 4 Department of Orthodontics and Dentofacial Orthopaedics, Hi-Tech Dental College and Hospital, Bhubaneswar, Odisha, India; 5 Department of Dentistry, Shri Krishna Medical College Hospital, Muzaffarpur, Bihar, India; 6 Department of Oral Medicine and Radiology, Thai Moogambigai Dental College and Hospital, Dr. MGR Educational and Research Institute, Chennai, Tamil Nadu, India

**Keywords:** Frontal sinus, gender identification, paranasal sinus, PNS views

## Abstract

Frontal sinus morphometry shows forensic promise for gender determination, yet paranasal sinus radiographs remain underexplored compared to CT-based assessments despite their accessibility. Therefore, it is of interest to evaluate frontal sinus dimensions (area, width, height) in 300 healthy adults (>20 years) using standardized paranasal sinus views with digital measurement techniques. Right frontal sinus height emerged as the most reliable predictor, achieving 69% accuracy for gender discrimination across all measured parameters. Paranasal radiographs demonstrated clinically viable predictive performance, particularly for height-based analysis compared with area- or width-based metrics. Thus, we document paranasal sinus views as a practical, cost-effective forensic tool for gender estimation when advanced imaging is unavailable.

## Background:

Use of radiology and various radiographs in forensics includes reporting, interpretation and performing radiographic procedures and assessments that involve the court and the law. Use of radiographs in forensics offers various benefits, including their quick, easy and simple nature. Radiographic methods are also financially beneficial compared with other commonly used techniques, including those that assess DNA [1, 2]. The frontal sinus is among the four paranasal sinuses that are seen in the confines of the frontal bone. It is beneficial in the forensic science field as it is unique in each subject and is a highly variable anatomic structure. It was first assessed post-mortem and antemortem in the year 1921 by Schuller on the radiographical views of the frontal sinus for the identification of a subject. It had opened avenues for various radiographic studies of the frontal sinuses [3, 4]. In most studies that assessed the frontal sinuses on radiographs in the past, CT scans, PA views, or Caldwell views were used to assess frontal sinus morphology. These data show a significant difference in the shape and size of the frontal sinuses between genders. The frontal sinus was found to be larger in male subjects than in females, highlighting its vital role and distinct characteristics in gender identification [5, 6]. There is limited evidence in the literature to assess the gender of a subject by assessing the morphometry of the frontal sinuses on the PNS (paranasal sinus) view, as most prior radiographic studies have focused on frontal sinus evaluation using PA or Caldwell views rather than PNS based morphometric analysis [7]. Therefore, it is of interest to radiographically assess the morphology of the frontal sinus in the determination of gender using the paranasal sinus view.

## Materials and Methods:

The present radiographic anatomical study was conducted to assess the morphology of the frontal sinus for gender determination using the paranasal sinus view. The study was conducted at the Department of Human Anatomy of the Institute during the defined study period. The study was conducted after obtaining Ethical approval from the Institutional Ethical Committee. Verbal and written informed consent was obtained from all subjects before the study. The study cohort comprised 300 adult healthy subjects aged 20 years or older, selected randomly, with equal numbers of males and females. The excluded subjects from the study were known intrinsic or extrinsic paranasal sinus diseases, endocrine disorders, systemic diseases, cranio-maxillofacial surgery history, previous orthodontic treatment and/ or history of trauma. After a comprehensive clinical assessment, a digital paranasal sinus view was obtained in all study subjects as a single count using standard techniques and technologies. The exposure parameters were also kept standardized at 17 seconds, 5 mA and 68 kV. The area, width and height of the frontal sinus were also digitally assessed using AutoCAD software. For standardization of the morphometric assessment of the sinus on the acquired images, a baseline was drawn on the radiographs at the base of the lune at the supraorbital ridge for the determination of the height of the frontal sinus. The margins on the radiograph were assessed to evaluate the maximum width of the sinus. The findings specific to the frontal sinuses were collected using a preformed pro forma made for the study. The collected data were recorded in the tables and statistically analyzed using logistic regression, Wilcoxon Signed Rank test, Mann-Whitney U test and Shapiro- Wilk test in SPSS (Statistical Package for Social Science) version 24.0 (IBM Corp., Armonk, NY, USA). The parameters considered were the frontal sinus area, maximum width and maximum height.

## Results:

The present radiographic anatomical study was conducted to assess the morphology of the frontal sinus for gender determination using the paranasal sinus view. The study considered 300 subjects, comprising 150 females and 150 males. It was recorded that among 300 subjects, no sinus or absent sinus, unilateral frontal sinus and bilateral frontal sinus were seen in 1.3% (n=4), 5.35% (n=16) and 93.3% (n=280) subjects, respectively. Of the 300 assessed radiographs, 20 PNS views were excluded because they had either an absent sinus or a unilateral sinus. The results indicated that larger frontal sinus dimensions were observed in male study subjects than in females on both the left and right sides. The mean left frontal sinus values in males were higher compared to the mean left frontal sinus values in females. The mean dimensions of area, width and height of the right frontal sinus were 508.65, 24.94 mm and 20.84 mm, respectively, whereas, for the left side, these dimensions were 620.13 mm^2^, 18.49 mm and 30.73 mm. The mean dimensions for the right side of the frontal sinus in females for area, width and height were 432.81 mm^2^, 22.03 mm and 16.91mm, respectively, whereas for the left side, these dimensions were 533.37 mm^2^, 15.26 mm and 27.77 mm (Table 1 - see PDF). Considering the left-sided sinus height, it was considerably greater than the right-sided frontal sinus height in the study (p < 0.05). The mean height of the left frontal sinus was 23.32±8.73 mm and on the right side of the sinus, the mean height was 18.94±6.43 mm. The width of the right side of the frontal sinus was significantly greater than that of the left side (p < 0.05), with mean widths of 23.53 ± 10.64 mm and 16.94 ± 6.34 mm, respectively. In the left sinus, the area was significantly greater than on the right side, with mean areas of 578.59±238.48 and 472.36±198.01 mm^2^, respectively, with p < 0.05 (Table 2 - see PDF). In the logistic regression analysis (Table 3 - see PDF), among all measured frontal sinus parameters, only the right side height showed a statistically significant association with sex (p=0.006), with an odds ratio of 0.86, indicating that higher right side sinus height was associated with a lower likelihood of the subject being female. None of the remaining parameters (right and left area, right and left width, left height) reached statistical significance (p>0.05). The constant term was statistically significant(p=0.001), suggesting that the model baseline had strong predictive value. Together, these findings indicate that right side frontal sinus height was the most reliable morphometric predictor for sex in the present study.

## Discussion:

The present study considered 300 subjects, comprising 150 females and 150 males. It was recorded that among 300 subjects, no sinus or absent sinus, unilateral frontal sinus and bilateral frontal sinus were seen in 1.3% (n=4), 5.35% (n=16) and 93.3% (n=280) subjects, respectively. Of the 300 assessed radiographs, 20 PNS views were excluded because they had either an absent sinus or a unilateral sinus. This data is consistent with previous studies by Shah *et al.* [8] in 2021 and Al Hatmi *et al.* [9] in 2023, in which comparable data were reported. The results of the present study indicated that, on both the left and right sides, male study subjects had larger frontal sinus dimensions than female subjects. The mean left frontal sinus values in males were higher compared to the mean left frontal sinus values in females. The mean dimensions of area, width and height of the right frontal sinus were 508.65 mm^2^, 24.94 mm and 20.84 mm, respectively, whereas, for the left side, these dimensions were 620.13 mm^2^, 18.49mm and 30.73mm. The mean dimensions for the right side of the frontal sinus in females were 432.81 mm^2^, 22.03 mm and 16.91mm, respectively, whereas for the left side, these dimensions were 533.37 mm^2^, 15.26 mm and 27.77 mm, respectively. These results correlated with the findings from Soman *et al.* [10] in 2016 and Belaldavar *et al.* [11] in 2014, in which the reported dimension results were comparable to those of this study. For the consideration of the left side sinus height, it was considerably greater than the right side of the frontal sinus in the study, with p<0.05. The mean height of the left frontal sinus was 23.32±8.73 mm and on the right side of the sinus, the mean height was 18.94±6.43 mm. The width of the right side of the frontal sinus was significantly greater than that of the left side (p < 0.05), with mean widths of 23.53 ± 10.64 mm and 16.94 ± 6.34 mm, respectively. In the left sinus, the area was significantly greater than on the right side, with mean areas of 578.59±238.48 mm^2^ and 472.36±198.01 mm^2^, respectively. These findings aligned with those of Mathur *et al.* [12] and Raoof *et al.* [13] in which the results for the width, height and area of the frontal sinus reported in this work were similar to those reported by the authors. For the use of logistic regression analysis in study parameters, the accuracy rate considered for the correct classification of area, width and height on the right side of the sinus was 58.4%, 63.4% and 62%, respectively and for the right side, these parameters were 57.7% and 64.1% for area and height, respectively. It was also noted that the width on the right side of the sinus was not a significant predictor for frontal sinus morphometry, with p>0.05. To determine whether a subject was male or female, the most reliable parameter was height on the right side of the frontal sinus. After correlating all study parameters, the accuracy for classifying a subject as female or male was 68.4%. These results aligned with the findings of Lee *et al.* [14] in 2010 and Ponde *et al.* [15] in 2003, in which the authors also reported that the most reliable parameter was height on the right side of the frontal sinus.

## Conclusion:

We show that dimensions of the frontal sinus evaluated from the paranasal sinus view can serve as a vital aid in determining gender in forensic cases. A larger number of subjects and long-term assessment will be helpful for better application of the results from the present study.
